# *Trans*-2-hexenal downregulates several pathogenicity genes of *Pseudogymnoascus destructans*, the causative agent of white-nose syndrome in bats

**DOI:** 10.1093/jimb/kuab060

**Published:** 2021-08-20

**Authors:** Victoria L Korn, Kayla K Pennerman, Sally Padhi, Joan W Bennett

**Affiliations:** Ionic Water Technologies, Hamilton, NJ 08609, USA; Joint Institute for Food Safety and Applied Nutrition, University of Maryland, College Park, MD 20742, USA; Department of Plant Biology, Rutgers University, The State University of New Jersey, New Brunswick, NJ 08901, USA; Department of Plant Biology, Rutgers University, The State University of New Jersey, New Brunswick, NJ 08901, USA

**Keywords:** *Pseudogymnoascus destructans*, *Trans*-2-hexenal, White-nose syndrome (WNS), Fungal virulence factors, Fumigation

## Abstract

White-nose syndrome is an emergent wildlife disease that has killed millions of North American bats. It is caused by *Pseudogymnoascus destructans*, a cold-loving, invasive fungal pathogen that grows on bat tissues and disrupts normal hibernation patterns. Previous work identified *trans*-2-hexenal as a fungistatic volatile compound that potentially could be used as a fumigant against *P. destructans* in bat hibernacula. To determine the physiological responses of the fungus to *trans*-2-hexenal exposure, we characterized the *P. destructans* transcriptome in the presence and absence of *trans*-2-hexenal. Specifically, we analyzed the effects of sublethal concentrations (5 μmol/L, 10 μmol/L, and 20 μmol/L) of gas-phase *trans*-2-hexenal of the fungus grown in liquid culture. Among the three treatments, a total of 407 unique differentially expressed genes (DEGs) were identified, of which 74 were commonly affected across all three treatments, with 44 upregulated and 30 downregulated. Downregulated DEGs included several probable virulence genes including those coding for a high-affinity iron permease, a superoxide dismutase, and two protein-degrading enzymes. There was an accompanying upregulation of an ion homeostasis gene, as well as several genes involved in transcription, translation, and other essential cellular processes. These data provide insights into the mechanisms of action of *trans*-2-hexenal as an anti-fungal fumigant that is active at cold temperatures and will guide future studies on the molecular mechanisms by which six carbon volatiles inhibit growth of *P. destructans* and other pathogenic fungi.

## Introduction

White-nose syndrome (WNS) is a fungal disease of hibernating bats that has devastated North American bat populations since its discovery in the winter of 2006–2007 (Blehert et al., 2009; Frick et al., 2010). WNS is caused by the psychrophilic species *Pseudogymnoascus destructans* (formerly *Geomyces destructans*) which grows optimally between 12.5°C and 15.8°C and infects bats while they are hibernating (Verant et al., 2012). The precipitous decline in bat populations has negatively affected ecosystem services provided by bats, especially their role in controlling insects that cause damage to agricultural crops (Boyles et al., 2011).

A concerted national research effort has sought to develop treatment methods to curb the growth of *P. destructans* and spans approaches that use a spectrum of biological, chemical, and physical control agents. For example, one promising biocontrol strategy uses *Pseudomonas* spp. isolated from the skin of bats that were found effective in constraining the growth of *P. destructans* in vitro (Hoyt et al., 2015). A subsequent extensive survey of over 350 bacteria and fungi isolated from bat hibernacula yielded a further 32 fungal and 60 bacterial isolates that showed biocontrol potential. Of these, the fungal species *Oidiodendron truncatum* produced several nontoxic norditerpene oidiolactones that were especially active against *P. destructans* (Rusman et al., 2020). Vaccines are yet another biologically based approach that have shown efficacy in preliminary trials (Rocke et al., 2019).

Under the category of physical agents, ultraviolet light is particularly promising for WNS control. *P. destructans* is far more sensitive to UV light than other *Pseudogymnoascus* species (Palmer et al., 2018) and shows practical promise for use in bat hibernacula (Kwait et al., 2021). Nevertheless, to date, most of the research on *P. destructans* growth inhibition has involved chemical control measures. Chemical treatments that have shown promise include azole drugs (Chaturvedi et al., 2011); chitosan (Kulhanek, 2016; Lopez-Moya et al., 2019); and linoleic acid (Frank et al., 2016). In addition, efficacy has been shown by exposure to a number of low-molecular-weight volatile organic compounds (VOCs) such as *trans, trans-*farnesol (Raudabaugh & Miller, 2015); 2-methyl-1-butanol, 2-methyl-1-propanol, and 1-pentanol (Micalizzi & Smith 2020), and mixtures of natural bacterial VOCs (Cornelison et al., 2014a, 2014b). In our laboratory, we have investigated possible fumigation treatments for bat hibernacula using environmentally safe VOCs and have reported that low concentrations of gas phase racemic 1-octen-3-ol (“mushroom alcohol”) can inhibit growth of *P. destructans* (Padhi et al., 2017). Furthermore, we found that the R enantiomer of 1-octen-3-ol was more effective than the S enantiomer, and that gas phase *trans*-2-hexenal (“leaf aldehyde”) was more effective than 1-octen-3-ol (Padhi et al., 2018). At a concentration of 0.05 μmol/ml, vapor phase *trans*-2-hexenal inhibited growth of mycelial plugs and prevented spore germination of *P. destructans* (Padhi et al., 2018). To our knowledge, this was one of the first studies to show that a volatile agent that is commonly used to control plant pathogenic fungi also shows activity against an animal pathogen.

Several laboratories have used molecular approaches to investigate the infection process during WNS. For instance, Field et al. (2015) showed that the pattern of mammalian gene expression associated with WNS in hibernating bats was accompanied by an innate antifungal immune response such as that caused by *Candida albicans* infections (Field et al., 2015). They identified several differences in fungal gene expression during the infective process, including expression of a number of *P. destructans* secreted proteases that serve as postulated virulence factors (Field et al., 2015). After the genome of *P. destructans* was sequenced and annotated (Drees et al., 2016), it opened the way for enhanced genetic analysis of the infection process and the response of *P. destructans* to different environmental agents. The effect of different nutrients and temperatures on the *P. destructans* transcriptome has shown that transcript levels were significantly different with different substrates and incubation temperatures, with substrate having a greater impact that temperature (Donaldson et al., 2018). Transcriptomics also can be used to compare whole transcriptome changes in gene expression between the pathogen alone, the pathogen infecting the host, and the host lacking the pathogen (Enguita et al., 2016). This approach was used by Reeder et al. (2017) who showed that the most significantly differentially expressed genes (DEGs) were involved in cell wall remodeling, heat shock response, and micronutrient acquisition. Herein, we have conducted a transcriptomic study to identify genes that are differentially expressed by *P. destructans* following exposure to *trans-*2-hexenal in order to better understand the inhibitory ability of this six-carbon VOC. The DEGs have been annotated to give putative identifications of their functions.

The immediate aim of our study is to identify fungal genes involved in the mode of action of *trans*-2-hexenal against *P. destructans*. The long-term goals of our research are to provide information that can be used to develop rational strategies for finding agents that may be used in the control of WNS, as well as to understand something of the critical fungal metabolic pathways affected by *trans*-2-hexenal, a natural metabolite produced by plants that is known to have a broad range of antifungal activity against plant pathogens.

## Materials and Methods

### Volatile Organic Compound

Liquid phase *trans-*2-hexenal (IUPAC name = (E)-hex-2-enal; synonyms include *trans-*2-hexen-1-al, (E)-2-hexenal, 3-propyl acrolein, and leaf aldehyde) was purchased as a chemical standard from Sigma-Aldrich (St. Louis, MO, USA). Aliquots of liquid were added in appropriate amounts such that after evaporation they delivered concentrations of 5, 10, or 20 μmol/L.

### Fungal Strain and Media

*P. destructans*, (MYA-4855TM) was obtained from the American Type Culture Collection, Manassas, VA, USA. Throughout our work, *P. destructans* was handled according to all procedures required for biosafety level 2 classification pathogens. Sabouraud Dextrose Agar (Difco, Becton Dickinson & Company, Sparks, MD) supplemented with 200 mg/L MnSO_4_ was used for the production of *P. destructans* conidiospores. Exposure experiments with *trans-*2-hexenal were conducted with *P. destructans* in Potato Dextrose Broth (PDB) (Difco).

### Conidiospore Production and Isolation

*P. destructans* conidiospores were harvested from plates that had been incubated at 15°C for 21 days. They were collected by adding 10 mL 0.05% (v/v) Tween 80 and 0.9% (w/v) NaCl solution to each plate and gently scraping the fungal growth with an inoculation loop to help release the spores. The suspension was then filtered through glass wool and the flowthrough was centrifuged at 5,000 rpm for 15 min. The supernatant was removed and the conidiospore pellet was resuspended in 10 mL of 0.2 M phosphate buffered saline solution (pH 7.0; Padhi et al., 2018). Viable counts of conidiospores were determined by plate dilution assay.

### Exposure of *P. destructans* to Gas Phase *Trans*-2-hexenal

Stationary cultures of *P. destructans* were prepared by inoculation with ∼ 2 × 10^5^ conidiospores. Wheaton 500 mL media bottles were used, each containing 100 mL of PDB. They were incubated for 4 weeks at 10°C and were then exposed to 0, 5, 10, or 20 μmol/L *trans*-2-hexenal by pipetting liquid *trans-*2-hexenal onto a 0.2 μm membrane which was taped to the inside of the cap of each bottle. Caps were slightly loosened to allow some airflow and then sealed with a single layer of Parafilm before incubation for an additional 4 weeks at 10°C. Each treatment was performed in triplicate.

### RNA Extraction and Sequencing

After 8 weeks of incubation, the loads of mycelia from each treated sample bottle were separated from the growth media by filtrating out the spent media. Using a sterile spatula, duplicate samples from each mycelial load were removed, weighed in cryogenic tubes, frozen in liquid nitrogen, and then crushed with a micro pestle before transfer to a bead bashing tube, followed by vortexing for 10 minutes. Using the ZR Fungal/Bacterial RNA Mini Prep kit (Zymo Research Corporation, Irvine, CA, USA), RNA was extracted according to the manufacturer's instructions and sent to the Genome Core facility at the Waksman Institute in Rutgers University for quality check, preparation, and sequencing. An Illumina NextSeq 500 instrument was used to yield single-end 1 × 75 bp reads. Sequencing reads without adapter sequences were deposited in the NCBI SRA database under accession number PRJNA523070.

### Bioinformatics Pipeline

The genome sequence and annotation files of *P. destructans* were retrieved from the NCBI Genome database (Drees et al., 2016) (assembly GCA_001641265.1). FastQC version 0.11.5 was used to check the quality of the sequencing reads before aligning them to the genome of *P. destructans* using STAR version 2.6 guided by the GFF3 annotation file (Dobin et al., 2013). The output SAM files were converted to sorted BAM files using Samtools version 1.7. Stringtie version 1.3.5 was used for differential gene expression analysis with DESeq2 version 1.22.1 (Li et al., 2009; Love et al., 2014; Pertea et al., 2015). To identify the most relevant transcripts for comparisons between control and experimental groups, an adjusted *p*-value ≤1^−5^ and a log_2_ fold change ≥1 or ≤–1 was considered statistically significant. Blast2GO was used to annotate such genes (Götz et al., 2008).

## Results

**Fig. 1.**
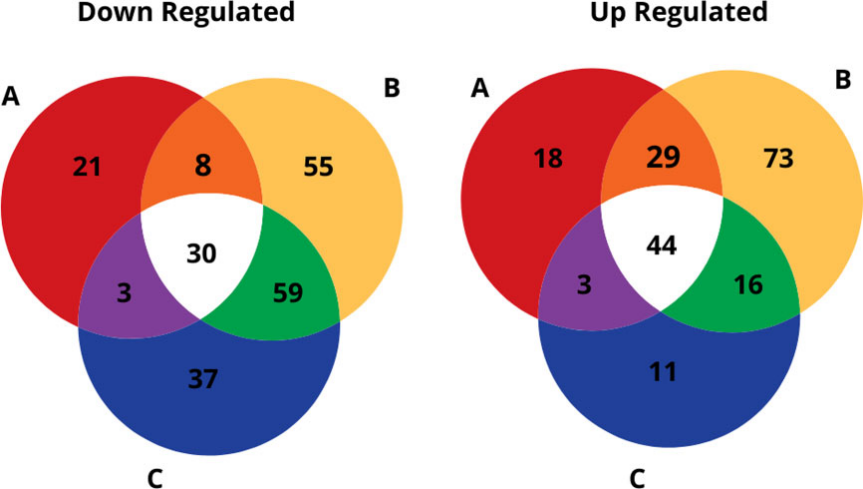
Venn diagrams of statistically significantly differentially expressed genes (DEGs) based on an adjusted *p*-value <1^−5^ and a log_2_ fold change <1^−5^ and a log_2_ fold change >1 or ←1 for both the down (left) and up (right) regulated genes for treatments of *trans*-2-hexenal at (A) 5 μmol/L, (B) 10 μmol/L, and (C) 20 μmol/L.

**Fig. 2.**
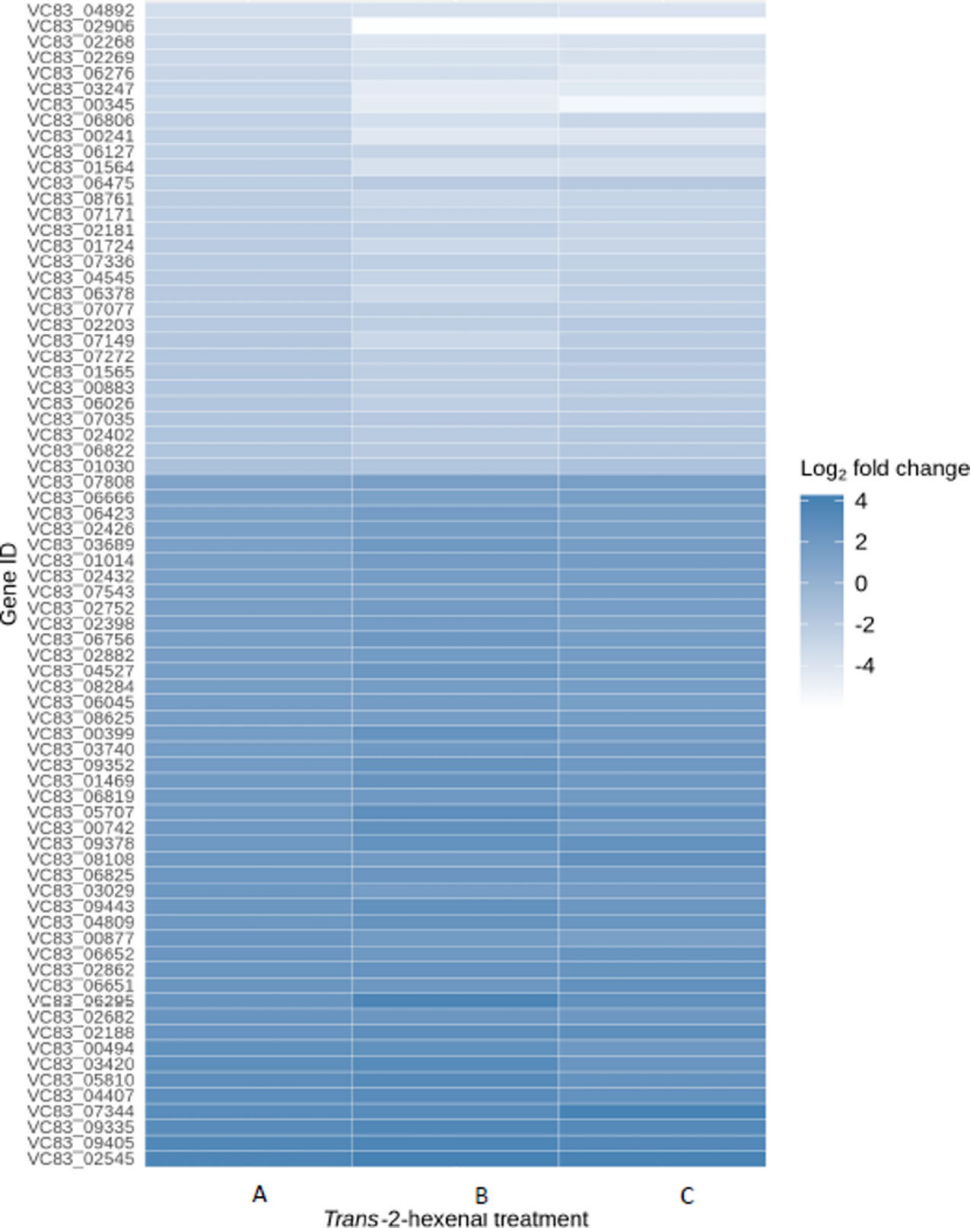
Heatmap of differentially expressed genes (DEGs) for (A) 5 μmol/L, (B) 10 μmol/L, and (C) 20 μmol/L gas phase *trans*-2-hexenal exposure. See Supplementary Tables S1 and S2 for functional annotations of genes.

Over >92% of the sequencing reads had quality scores >Q30 for all treatment replicates. We identified the statistically significant DEGs that had an adjusted *p*-value ≤1^−5^ and a log_2_ fold change ≥1 or ≤–1. Of the three sublethal levels of *trans*-2-hexenal tested, the highest number of DEGs (314) was observed after exposure at 10 μmol/L. Across all three treatments, there were 407 unique DEGs that responded to exposure to the three tested levels of *trans*-2-hexenal exposure of which 194 were upregulated and 213 downregulated (Fig. 1). Of these unique DEGs, 74 were common among all treatments, of which 44 were upregulated and 30 were downregulated. A heat map showing the 74 commonly expressed, unique DEGs and their gene IDs is given in Fig. 2. Supplementary Tables S1 and S2 list the individual genes, their annotations, and their fold changes in response to the three levels of *trans*-2-hexenal.

When initially checked against the NCBI database, more than half of the unique DEGs did not have a functional annotation and were described as hypothetical proteins. Further analysis with Blast2GO provided 23 more homologies. Nevertheless, at the end of our analysis, 23 of the upregulated DEGs and 10 of the downregulated DEGs remained categorized as “hypothetical proteins.” Several of the commonly upregulated genes were involved in transcription, translation, and replication, such as U3 small nucleolar RNA-associated protein 13 (VC83_02882) (Dragon et al., 2002); translation initiation factor eIF4A (VC83_04809) (Hernández & Vazquez-Pianzola, 2005); protein kinase Rio1 (VC83_06045) (Guderian et al., 2011); translational elongation factor EF-1 alpha (VC83_06666) (Song et al., 1989); DEAH-box ATP-dependent RNA helicase prp43 (VC83_08625) (Arenas & Abelson, 1997); ATP-dependent RNA helicase *dbp7* (VC83_06819) (Daugeron & Linder, 1998); eukaryotic translation initiation factor 6 (VC83_00399) (Russell & Spremulli, 1980); ribosome-binding protein (VC83_06825) (Ho & Johnson, 1999; Niessing, 2012); and DNA-dependent ATPase of the nucleotide excision repair factor 4 complex (VC83_04407) (Guzder et al., 1998). Other upregulated genes included those predicted to code for a mitochondrial glycerol-3-phosphate dehydrogenase (VC83_03689) (Rønnow & Kielland-Brandt, 1993); the Rab protein geranylgeranyl transferase component A (VC83_02426) that aids in vesicle transport as a transport protein (Andres et al., 1993); a magnesium transporter (VC83_07808) (Lee & Gardner, 2006); and a calcium-transporting ATPase 2 (VC83_01014) that acts as a part of ion homeostasis and can contribute to survival (Reeder et al., 2017).

Downregulated genes included those predicted to code for glyceraldehyde-3-phosphate dehydrogenase 1 (VC83_08761), which catalyzes the sixth step of glycolysis (Harris & Waters, 1976); phosphomevalonate kinase (VC83_06822), which is essential in isoprenoid/sterol biosynthesis (Tsay & Robinson, 1991); methylglyoxal reductase (NADPH-dependent) *gre2* (VC83_ 00883), which is involved in magnesium utilization, reducing methylglyoxal to (S)-lactaldehyde (Murata et al., 1985); and SED4, involved in vesicle formation and transport at the endoplasmic reticulum (VC83_06276) (Watson & Stephens, 2005).

The most notable of the downregulated genes were those that were implicated as fungal virulence factors in *P. destructans* or other fungal pathogens. These include SOD1 superoxide dismutase (VC83_07077), which is essential to removing toxic superoxide radicals by creating molecular oxygen (McCord & Fridovich, 1969) and a high-affinity iron permease (VC83_07149), associated with pathogenicity in *C. albicans* and *Rhizopus oryzae* (Ramanan & Wang, 2000). In addition, the following genes associated with protein metabolism were downregulated: peptidase S28 (VC83_03247), a serine peptidase (Barrett, 1994); as well as an amino acid permease (VC83_06026); a high-affinity iron permease (VC83_07194), and the subtilisin-like protease 1 gene (VC83_04892) which codes for a collagen degradation enzyme and was named PsSP1 by Pannkuk et al. (2015) and Destructin-2 by O'Donoghue et al. (2015).

## Discussion

Agricultural scientists have adapted several natural volatile compounds as postharvest fumigants to control plant pathogenic fungi in stored fruits and vegetables (Tripathi & Dubey 2004; Kudalkar et al., 2012; Mari et al., 2016). Several six-carbon volatiles display antifungal effectiveness under cold storage conditions. These biogenic VOCs have limited toxicity in mammals (Akutsu et al., 2002; Ito et al., 2009) and a degree of volatility that allows their application in refrigerated storage facilities (Jongen 2005). Because fungal plant and animal pathogens often share physiological features, and because refrigerated storage facilities and bat hibernacula are cognate environmental conditions, volatile agents offer promise against *P. destructans* in WNS control. We focused on *trans*-2-hexenal, a compound that is both commercially available and a generally recognized as safe (GRAS) food additive by the U.S. Food and Drug Administration (FDA 2018). It is found in olive oil (Kubo et al., 1995) and is characteristic of the odor of cut grass where it is commonly called a “green leaf aldehyde” and theorized to be involved in plant defense against insect herbivores in nature (Paré & Tumlinson, 1999). Vapors of *trans-*2-hexenal inhibit plant pathogenic fungi including *Aspergillus flavus* growth in corn (De Lucca et al., 2011), *Penicillium expansum* on pears (Neri et al., 2006) and two species of *Fusarium* on wheat-chick pea rotations (Cruz et al., 2012). Our preliminary studies demonstrated that low concentrations of gas phase *trans*-2-hexenal also inhibited growth of *P. destructans* (Padhi et al., 2018). We hypothesized that exposure of *P. destructans* mycelia to *trans*-2-hexenal would cause changes in gene expression that might reveal genetic susceptibilities of this psychrophilic fungal pathogen. Because *P. destructans* is slow growing, we precultured the fungus for 4 weeks to obtain sufficient mycelium for transcriptomics analysis before exposing the cultures to three sublethal concentrations of vapors of *trans*-2-hexenal vapors and then continuing the incubation for another 4 weeks. Our goal was to use the analysis of the differential transcripts to help pinpoint the molecular mechanisms that are essential to pathogen growth and virulence, and guide future research to formulate precise methods for the control of WNS. We also hoped to shed light on the global biochemical processes that change in fungal pathogens in the presence of *trans*-2-hexenal.

In our study, across the three treatments with sublethal concentrations of *trans*-2-hexanal, a total of 407 statistically significant DEGs were identified, of which 74 were common across all three treatments. Of these common DEGs, 33 putative proteins identified in our analysis had insufficient sequence similarity to known proteins in current databases to generate a functional prediction and were scored as hypothetical (Supplementary Tables S1 and S2). The high number of hypothetical proteins (approx. 44%) provides evidence that many uncharacterized genes play important physiological roles in fungal responses to gas phase *trans*-2-hexenal.

Among the downregulated DEGs that were successfully annotated to known proteins, several were identified as having putative virulence functions. These include a high-affinity iron permease (VC83_07149), associated with pathogenicity in *C. albicans* and *R. oryzae* (Ramanan & Wang, 2000); as well as superoxide dismutase (VC83_07077). It is well known that mammalian innate immune cells produce reactive oxygen species (ROS) in order to destroy invading pathogens. In response, fungal pathogens use superoxide dismutase to detoxify ROS thereby evading host immune surveillance. Superoxide dismutases are documented virulence factors for a number of important fungal pathogens including *C. albicans, Cryptococcus neoformans, and Aspergillus fumigatus* (Frohner et al., 2009; Lambou et al., 2010).

With respect to *P. destructans* and WNS, proteases have received the greatest attention as putative virulence factors. Certain collagen degradation enzymes contribute to the pathogenicity in some pathogenic bacteria such as *Streptococcus* (Bonifait et al., 2010) and are similar to cuticle degrading enzymes that had previously been identified from fungi that parasitize insects (Yang et al, 2007). In 2015, a subtilisin-like serine protease (PdSP1) was identified as a major protease secreted by *P. destructans* grown in liquid culture. The researchers postulated that this collagen degrading enzyme might serve as a virulence factor involved in epidermal wing necrosis in WNS. Moreover, they found two similar proteins with high sequences homology to PsSP1 that they termed PsSP2 and PsSP3 (Pannkuk et al., 2015). Nearly simultaneously, another group doing similar work on the secretome of *P. destructans* isolated a serine endopeptidase that they named Destructin-1. Biochemical analysis of a recombinant form of Destructin-1 indicated that this enzyme efficiently degraded collagen. Another endopeptidase that showed 90% amino acid identity was isolated and named Destructin-2, and a third endopeptidase with 56% identity to Destructin-1 was named Destructin-3 (O'Donoghue et al., 2015). Only Destructin-2 (VC83_04892) had enriched transcript levels in the in vivo pathogenic context compared with the nonpathogenic conditions (Donaldson et al., 2018). The nomenclature for these peptidases is confusing because PdSP1 is synonymous with Destructin-2 and PdSP2 is synonymous with Destructins-1. A third protease from *P. destructans* that has been experimentally characterized is the serine peptidase PdCP1. It is an ortholog of tripeptide amino peptidase Sed2/SedB, which is a putative virulence factor from *A. fumigatus* (Reichard et al., 2006). Of the three proteases that have been experimentally characterized, in our study, only the gene for PdSP1 (Destructans-2) was downregulated.

Using a dual RNA Seq approach, Reeder et al. (2017) compared the changes in fungal gene expression that accompanied a transition from abiotic growth in culture to parasitic growth during WNS. As compared to gene expression of *P. destructans* grown in culture, they unexpectedly found significantly lower expression of the genes for the subtilisin family of proteases during infection (Reeder et al., 2017). Finally, and also perplexingly, the transcriptomics study by Davy et al. (2020), suggested that the production of virulence factors and increases in active biomass by *P. destructans* were similar in lesions on bats that were of varying susceptibilities. They conducted a transcriptomic analysis of *P. destructans* growing in lesion-positive and lesion-negative bat wing tissue. *P. destructans* responded similarly to growth lesions on diverse bat species *Myotis lucifugus, Eptesicus fuscus*, and *Myotis myotis*. Expression of the subtilisin-like serine proteases Destructin-1, -2, and -3 varied among the species with the greatest expression observed on the WNS-tolerant bat species *M. myotis* (Davy et al., 2020).

While we recognize that transcriptome analyses provide only a “snapshot” of gene expression, they nevertheless can illuminate a more context-dependent understanding of host–pathogen relationships. Our study suggests that the presence of *trans-*2-hexenal, a safe volatile known to inhibit growth of plant pathogenic fungi, causes the downregulation of a number of genes believed to be involved in virulence of *P. destructans*. To our knowledge, this is the first transcriptomics study of the effect of *trans-*2-hexenal on a fungus. Fumigation with natural VOCs has potential applications not only for plant pathogenic fungi, but in wildlife disease scenarios outside of WNS. Information about the mechanistic basis of the fungistatic and fungicidal effects of volatile agents can guide future research that seeks to translate these basic findings into practical methods for the control of a wide variety of fungal pathogens.

## Supplementary Material

kuab060_Supplemental_File

## References

[bib1] Akutsu H. , Kikusui T., Takeuchi Y., Sano K., Hatanaka A., Mori Y. (2002). Alleviating effects of plant-derived fragrances on stress-induced hyperthermia in rats. Physiology & Behavior, 75, 355–360. 11897262 10.1016/s0031-9384(01)00670-9

[bib2] Andres D. A. , Seabra M. C., Brown M. S., Armstrong S. A., Smeland T. E., Cremers F. P., Goldstein J. L. (1993). cDNA cloning of component A of Rab geranylgeranyl transferase and demonstration of its role as a Rab escort protein. Cell, 73(6), 1091–1099. 8513495 10.1016/0092-8674(93)90639-8

[bib3] Arenas J. E. , Abelson J. N. (1997). Prp43: An RNA helicase-like factor involved in spliceosome disassembly. Proceedings of the National Academy of Sciences of the United States of America, 94(22), 11798–11802. 9342317 10.1073/pnas.94.22.11798 PMC23592

[bib4] Barrett A. J. (1994). Classification of peptidases. Methods in Enzymology, 244, 1–15. 7845199 10.1016/0076-6879(94)44003-4

[bib5] Blehert D. S. , Hicks A. C., Behr M., Meteyer C. U., Berlowski-Zier B. M., Buckles E. L., Coleman J. T. H., Darling S. R., Gargas A., Niver R., Okoniewski J. C., Rudd R. J., Stone W. B. (2009). Bat white-nose syndrome: An emerging fungal pathogen? Science, 323(5911), 227. 18974316 10.1126/science.1163874

[bib6] Bonifait L. , de la Cruz, Dominguez-Punaro M., Vaillancourt K., Bart C., Slater J., Frenette M., Gottschalk M., Grenier D. (2010). The cell envelope subtilisin-like proteinase is a virulence determinant for *Streptococcus suis*. BMC Microbiology, 10(1), 42. 10.1186/1471-2180-10-42. 20146817 PMC2832634

[bib7] Boyles J. G. , Cryan P. M., McCrcken G. F., Kunz T. H. (2011). Economic importance of bats in agriculture. Science, 332(6025), 41–42. 21454775 10.1126/science.1201366

[bib9] Chaturvedi S. , Rajkumar S. S., Li X., Hurteau G. J., Shtutman M., Chaturvedi V. (2011). Antifungal testing and high-throughput screening of compound library against *Geomyces destructans*, the etiologic agent of geomycosis (WNS) in bats. PLoS One, 6(3), e17032. 21399675 10.1371/journal.pone.0017032 PMC3047530

[bib10] Cornelison C. T. , Gabriel K. T., Barlament C., Crow S. A. Jr. (2014a). Inhibition of *Pseudogymnoascus destructans* growth from conidia and mycelial extension by bacterially produced volatile organic compounds. Mycopathologia, 177(1-2), 1–10. 24190516 10.1007/s11046-013-9716-2

[bib11] Cornelison C. T. , Keel M. K., Gabriel K. T., Barlament C. K., Tucker T. A., Pierce G. E., Crow S. A. (2014b). A preliminary report on the contact-independent antagonism of *Pseudogymnoascus destructans* by *Rhodococcus rhodochrous* strain DAP96253. BMC Microbiology, 14(1), 246. 25253442 10.1186/s12866-014-0246-y PMC4181622

[bib12] Cruz A. F. , Hamel C., Yang C., Matsubara T., Gan Y., Singh A. K., Kuwada K., Ishii T. (2012). Phytochemicals to suppress *Fusarium* head blight in wheat-chickpea rotation. Phytochemistry, 78, 72–80. 22520499 10.1016/j.phytochem.2012.03.003

[bib14] Davy C. M. , Donaldson M. E., Bandouchova H., Breit A. M., Dorville N., Dzal Y. A., Kovacova V., Kunkel E. L., Martínková N., Norquay K., Paterson J. E., Zukal J., Pikula J., Willis C., Kyle C. J. (2020). Transcriptional host-pathogen responses of *Pseudogymnoascus destructans* and three species of bats with white-nose syndrome. Virulence, 11(1), 781–794. 32552222 10.1080/21505594.2020.1768018 PMC7549942

[bib15] Daugeron M. C. , Linder P. (1998). Dbp7p, a putative ATP-dependent RNA helicase from Saccharomyces cerevisiae, is required for 60S ribosomal subunit assembly. Rna, 4(5), 566–581. 9582098 10.1017/s1355838298980190 PMC1369640

[bib16] De Lucca A. J. , Carter-Wientjes C. H., Boué S., Bhatnagar D. (2011). Volatile trans-2-hexenal, a soybean aldehyde, inhibits *Aspergillus flavus* growth and aflatoxin production in corn. Journal of Food Science, 76(6), M381–M386. 22417509 10.1111/j.1750-3841.2011.02250.x

[bib17] Dobin A. , Davis C. A., Schlesinger F., Drenkow J., Zaleski C., Jha S., Batut P., Chaisson M., Gingeras T. R. (2013). STAR: Ultrafast universal RNA-seq aligner. Bioinformatics, 29(1), 15–21. 23104886 10.1093/bioinformatics/bts635 PMC3530905

[bib18] Donaldson M. E. , Davy C. M., Vanderwolf K. J., Willis C. K. R., Saville B. J., Kyle C. J. (2018). Growth medium and incubation temperature alter the *Pseudogymnoascus destructans* transcriptome: Implications in identifying virulence factors. Mycologia, 110(2), 300–315. 29737946 10.1080/00275514.2018.1438223

[bib19] Dragon F. , Gallagher J. E. G., Compagnone-Post P. A., Mitchell B. M., Porwancher K. A., Wehner K. A., Wormsley S., Settlage R. E., Shabanowitz J., Osheim Y., Beyer A. L., Hunt D. F., Baserga S. J. (2002). A large nucleolar U3 ribonucleoprotein required for 18S ribosomal RNA biogenesis. Nature, 417(6892), 967–970. 12068309 10.1038/nature00769 PMC11487672

[bib20] Drees K. P. , Palmer J. M., Sebra R., Lorch J. M., Chen C., Wu C.-C., Bok J. W., Keller N. P., Blehert D. S., Cuomo C. A., Lindner D. L., Foster J. T. (2016). Use of multiple sequencing technologies to produce a high-quality genome of thefFungus *Pseudogymnoascus destructans*, the causative agent of bat White-Nose Syndrome. Genome Announcements, 4(3), e00445–16. 27365344 10.1128/genomeA.00445-16 PMC4929507

[bib21] Enguita F. J. , Costa M. C., Fusco-Ameida A. M., Mendes-Giannini M. J., Leitão A. L. (2016). Transcriptomic crosstalk between fungal invasive pathogens and their host cells: Opportunities and challenges for next-generation sequencing methods. Journal of Fungi, 2(1), 7. 10.3390/jof2010007. PMC5753088 29376924

[bib22] Field K. A. , Johnson J. S., Lilley T. M., Reeder S. M., Rogers E. J., Behr M. J., Reeder D. M. (2015). The white-nose syndrome transcriptome: Activation of anti-fungal host responses in wing tissue of hibernating little brown *Myotis*. PLoS Pathogens, 11(10), e1005168. 10.1371/journal.ppat.1005168. 26426272 PMC4591128

[bib23] Food and Drug Administration . (2018). Substances added to foods. https://www.fda.gov/food/food-additives-petitions/substances-added-food-formerly-eafus.

[bib24] Frank C. L. , Ingala M. R., Ravenelle R. E., Dougherty-Howard K., Wicks S. O., Herzog C., Rudd R. J. (2016). The effects of cutaneous fatty acids on the growth of *Pseudogymnoascus destructans*, the etiological agent of White-Nose Syndrome (WNS). PLoS One, 11(4), e0153535. 27070905 10.1371/journal.pone.0153535 PMC4829186

[bib25] Frick W. F. , Pollock J. F., Hicks A. C., Langwig K. E., Reynolds D. S., Turner G. G., Butchkoski C. M., Kunz T. H. (2010). An emerging disease causes regional population collapse of a common North American bat species. Science, 329(5992), 679–682. 20689016 10.1126/science.1188594

[bib26] Frohner I. E. , Bourgeois C., Yatsyk K., Majer O., Kuchler K. (2009). *Candida albicans* cell surface superoxide dismutases degrade host-derived reactive oxygen species to escape innate immune surveillance. Molecular Microbiology, 71(1), 240–252. 19019164 10.1111/j.1365-2958.2008.06528.x PMC2713856

[bib27] Götz S. , García-Gómez J. M., Terol J., Williams T. D., Nagaraj S. H., Nueda M. J., Robles M., Talón M., Dopazo J., Conesa A. (2008). High-throughput functional annotation and data mining with the Blast2GO suite. Nucleic Acids Research, 36(10), 3420–3435. 18445632 10.1093/nar/gkn176 PMC2425479

[bib29] Guderian G. , Peter C., Wiesner J., Sickmann A., Schulze-Osthoff K., Fischer U., Grimmler M. (2011). RioK1, a new interactor of protein arginine methyltransferase 5 (PRMT5), competes with pICln for binding and modulates PRMT5 complex composition and substrate specificity. The Journal of Biological Chemistry, 286(3), 1976–1986. 21081503 10.1074/jbc.M110.148486 PMC3023494

[bib30] Guzder S. N. , Sung P., Prakash L., Prakash S. (1998). The DNA-dependent ATPase activity of yeast nucleotide excision repair factor 4 and its role in DNA damage recognition. The Journal of Biological Chemistry, 273(11), 6292–6296. 9497356 10.1074/jbc.273.11.6292

[bib31] Harris J. I. , Waters M. (1976). Glyceraldehyde-3-phosphate dehydrogenase. The Enzymes, 13, 1–49.

[bib32] Hernández G. , Vazquez-Pianzola P. (2005). Functional diversity of the eukaryotic translation initiation factors belonging to eIF4 families. Mechanisms of Development, 122(7-8), 865–876. 15922571 10.1016/j.mod.2005.04.002

[bib33] Ho J. H. -N. , Johnson A. W. (1999). NMD3 encodes an essential cytoplasmic protein required for stable 60S ribosomal subunits in *Saccharomyces cerevisiae*. Molecular and Cellular Biology, 19(3), 2389–2399. 10022925 10.1128/mcb.19.3.2389 PMC84031

[bib34] Hoyt J. R. , Cheng T. L., Langwig K. E., Hee M. M., Frick W. F., Kilpatrick A. M. (2015). Bacteria isolated from bats inhibit the growth of *Pseudogymnoascus destructans*, the causative agent of white-nose syndrome. PLoS One, 10(4), e0121329. 25853558 10.1371/journal.pone.0121329 PMC4390377

[bib36] Ito A. , Miyoshi M., Ueki S., Fukada M., Komaki R., Watanabe T. (2009). “Green odor” inhalation by rats down-regulates stress-induced increases in Fos expression in stress-related forebrain regions. Neuroscience Research, 65(2), 166–174. 19563846 10.1016/j.neures.2009.06.012

[bib37] Jongen W., Ed. (2005). Improving the safety of fresh fruit and vegetables. Woodhead Pub. Limited, Cambridge, England and CRC Press.

[bib38] Kulhanek T. C. (2016). The application of chitosan on an experimental infection of *Pseudogymnoascus destructans* increases survival in little brown bats. Western Michigan University Theses, 1–77.

[bib39] Kubo A. , Lunde C. S., Kubo I. (1995). Antimicrobial activity of the olive oil flavor compounds. Journal of Agricultural and Food Chemistry, 43(6), 1629–1633.

[bib39a] Kudalkar P. , Strobel G., Riyaz-Ul-Hassan S., Geary B., Sears J. (2012). Muscodor sutura, a novel endophytic fungus with volatile antibiotic activities. Mycoscience, 53(4), 319–325.

[bib40] Kwait R. , Herzog C., Kerwin K., Bennett J., Padhi S., Maslo B. (2021). Whole-room ultraviolet sanitization as a method for the site-level treatment of *Pseudogymnoascus destructans*. (under review).

[bib41] Lambou K. , Lamarre C., Beau R., Dufour N., Latge J. -P. (2010). Functional analysis of the superoxide dismutase family in *Aspergillus fumigatus*. Molecular Microbiology, 75(4), 910–923. 20487287 10.1111/j.1365-2958.2009.07024.x

[bib42] Lee J. -M. , Gardner R. C. (2006). Residues of the yeast ALR1 protein that are critical for magnesium uptake. Current Genetics, 49(1), 7–20. 16328501 10.1007/s00294-005-0037-y

[bib43] Li H. , Handsaker B., Wysoker A., Fennell T., Ruan J., Homer N., Marth G., Abecasis G., Durbin R. (2009). The sequence alignment/map format and SAMtools. Bioinformatics, 25(16), 2078–2079. 19505943 10.1093/bioinformatics/btp352 PMC2723002

[bib44] Lopez-Moya F. , Suarez-Fernandez M., Lopez-Llorca L. V. (2019). Molecular mechanisms of chitosan interactions with fungi and plants. International Journal of Molecular Sciences, 20(2), 332. 10.3390/ijms20020332 PMC6359256 30650540

[bib45] Love M. I. , Huber W., Anders S. (2014). Moderated estimation of fold change and dispersion for RNA-seq data with DESeq2. Genome Biology, 15(12), 550. 25516281 10.1186/s13059-014-0550-8 PMC4302049

[bib46] McCord J. M. , Fridovich I. (1969). Superoxide dismutase: An enzymatic function for erythrocuprein (hemocuprein). The Journal of Biological Chemistry, 244(22), 6049–6055. 5389100

[bib47] Mari M. , Bautista-Baños S., Sivakumar D. (2016). Decay control in the postharvest system: Role of microbial and plant volatile organic compounds. Postharvest Biology & Technology, 22, 70–81.

[bib48] Micalizzi E. W. , Smith M. L., 2020. Volatile organic compounds kill the white-nose syndrome fungus, *Pseudogymnoascus destructans*, in hibernaculum sediment. Canadian Journal of Microbiology, 66(10), 593–599. 32485113 10.1139/cjm-2020-0071

[bib49] Murata K. , Fukuda Y., Simosaka M., Watanabe K., Saikusa T., Kimura A. (1985). Metabolism of 2-oxoaldehyde in yeasts. Purification and characterization of NADPH-dependent methylglyoxal-reducing enzyme from *Saccharomyces cerevisiae*. European Journal of Biochemistry, 151(3), 631–636. 3896793 10.1111/j.1432-1033.1985.tb09151.x

[bib50] Neri F. , Mari M., Menniti A., Brigati S. (2006). Activity of trans-2-hexenal against *Penicillium expansum* in 'Conference' pears. Journal of Applied Microbiology, 100(6), 1186–1193. 16696666 10.1111/j.1365-2672.2006.02873.x

[bib51] Niessing D. (2012). RNA-Binding Proteins in Fungi and Their Role in mRNA Localization. RNA Binding Proteins, 81.

[bib52] O'Donoghue A. J. , Knudsen G. M., Beekman C., Perry J. A., Johnson A. D., DeRisi J. L., Craik C. S., Bennett R. J. (2015). Destructin-1 is a collagen-degrading endopeptidase secreted by *Pseudogymnoascus destructans*, the causative agent of white-nose syndrome. Proceedings of the National Academy of Sciences, 112(24), 7478–7483. 10.1073/pnas.1507082112 PMC4475985 25944934

[bib53] Padhi S. , Dias I., Bennett J. W. (2017). Two volatile-phase alcohols inhibit growth of Pseudogymnoascus destructans, causative agent of white-nose syndrome in bats. Mycology, 8(1), 11–16.

[bib54] Padhi S. , Dias I., Korn V. L., Bennett J. W. (2018). Pseudogymnoascus destructans: Causative agent of white-nose syndrome in bats is inhibited by safe volatile organic compounds. Journal of Fungi, 4(2), 48. 10.3390/jof4020048 PMC6023378 29642609

[bib55] Palmer J. M. , Drees K. P., Foster J. T., Lindner D. L. (2018). Extreme sensitivity to ultraviolet light in the fungal pathogen causing white-nose syndrome of bats. Nature Communications, 9. 10.1038/s41467-017-02441-z PMC5750222 29295979

[bib56] Pannkuk E. L. , Risch T. S., Savary B. J. (2015). Isolation and identification of a 712 extracellular subtilisin-like serine protease secreted by the bat pathogen Pseudogymnoascus destructans. PLoS One, 10(3), e0120508. 25785714 10.1371/journal.pone.0120508 PMC4364704

[bib57] Paré P. W. , Tumlinson J. H. (1999). Plant volatiles as a defense against insect herbivores. Plant Physiology, 121(2), 325–332. 10517823 PMC1539229

[bib58] Pertea M. , Pertea G. M., Antonescu C. M., Chang T. -C., Mendell J. T., Salzberg S. L. (2015). StringTie enables improved reconstruction of a transcriptome from RNA-seq reads. Nature Biotechnology, 33(3), 290–295. 10.1038/nbt.3122 PMC4643835 25690850

[bib59] Ramanan N. , Wang Y. (2000). A high-affinity iron permease essential for *Candida albicans* virulence. Science, 288(5468), 1062–1064. 10807578 10.1126/science.288.5468.1062

[bib60] Raudabaugh D. B. , Miller A. N. (2015). Effect of trans, trans-farnesol on *Pseudogymnoascus destructans* and several closely related species. Mycopathologia, 180(5-6), 325–332. 26162644 10.1007/s11046-015-9921-2

[bib61] Reeder S. M. , Palmer J. M., Prokkola J. M., Lilley T. M., Reeder D. M., Field K. A. (2017). *Pseudogymnoascus destructans* transcriptome changes during white-nose syndrome infections. Virulence, 8(8), 1695–1707. 28614673 10.1080/21505594.2017.1342910 PMC5810475

[bib62] Reichard U. , Léchenne B., Asif A. R., Streit F., Grouzmann E., Jousson O., Monod M. (2006). Sedolisins, a new class of secreted proteases from *Aspergillus fumigatus* with endoprotease or tripeptidyl-peptidase activity at acidic pHs. Applied and Environmental Microbiology, 72(3), 1739–1748. 16517617 10.1128/AEM.72.3.1739-1748.2006 PMC1393174

[bib63] Rønnow B. , Kielland-Brandt M. C. (1993). GUT2, a gene for mitochondrial glycerol 3-phosphate dehydrogenase of *Saccharomyces cerevisiae*. Yeast, 9(10), 1121–1130. 8256521 10.1002/yea.320091013

[bib64] Rocke T. E. , Kingstad-Bakke B., Wüthrich M., Stading B., Abbott R. C., Isidoro-Ayza M., Dobson H. E., dos Santos Dias L., Galles K., Lankton J. S., Falendysz E. A., Lorch J. M., Fites J. S., Lopera-Madrid J., White J. P., Klein B., Osorio J. E. (2019). Virally-vectored vaccine candidates against white-nose syndrome induce anti-fungal immune response in little brown bats (*Myotis lucifugus*). Scientific Reports, 9(1), 6788. 31043669 10.1038/s41598-019-43210-w PMC6494898

[bib65] Russell D. W. , Spremulli L. L. (1980). Mechanism of action of the wheat germ ribosome dissociation factor: Interaction with the 60 S subunit. Archives of Biochemistry and Biophysics, 201(2), 518–526. 6901609 10.1016/0003-9861(80)90540-8

[bib66] Rusman Y. , Wilson M. B., Williams J. M., Held B. W., Blanchette R. A., Anderson B. N., Lupfer C. R., Salomon C. E. (2020). Antifungal norditerpene oidiolactones from the fungus *Oidiodendron truncatum*, a potential biocontrol agent for White-Nose Syndrome in bats. Journal of Natural Products, 83(2), 344–353. 31986046 10.1021/acs.jnatprod.9b00789

[bib67] Song J. M. , Picologlou S., Grant C. M., Firoozan M., Tuite M. F., Liebman S. (1989). Elongation factor EF-1 alpha gene dosage alters translational fidelity in *Saccharomyces cerevisiae*. Molecular and Cellular Biology, 9, 4571–4575. 2685557 10.1128/mcb.9.10.4571-4575.1989 PMC362547

[bib68] Tripathi P. , Dubey N. K. (2004). Exploitation of natural products as an alternative strategy to control postharvest fungal rotting of fruit and vegetables. Postharvest Biology and Technology Journal, 32(3), 235–245.

[bib69] Tsay Y. H. , Robinson G. W. (1991). Cloning and characterization of ERG8, an essential gene of *Saccharomyces cerevisiae* that encodes phosphomevalonate kinase. Molecular and Cellular Biology, 11, 620–631. 1846667 10.1128/mcb.11.2.620-631.1991 PMC359713

[bib71] Verant M. L. , Boyles J. G., Waldrep W. Jr, Wibbelt G., Blehert D. S. (2012). Temperature-dependent growth of *Geomyces destructans*, the fungus that causes bat white-nose syndrome. PLoS One, 7(9), e46280. 23029462 10.1371/journal.pone.0046280 PMC3460873

[bib72] Watson P. , Stephens D. J. (2005). ER-to-Golgi transport: Form and formation of vesicular and tubular carriers. Biochimica et Biophysica Acta, 1744(3), 304–315. 15979504 10.1016/j.bbamcr.2005.03.003

[bib72a] Yang K. , Tian B., Liang L., Zhang K. (2007). Extracellular enzymes and the pathogenesis of nematophagous fungi. Applied Microbiology Biotechnology, 75, 21–31. 10.1007/s00253-007-0881-4 17318531

