# Supplementary material for: Trans-2-hexenal downregulates several pathogenicity genes of Pseudogymnoascus destructans, the causative agent of white-nose syndrome in bats
Source: J Ind Microbiol Biotechnol. 2021 Aug 20;48(9-10):kuab060. doi: 10.1093/jimb/kuab060 (PMC8788850; doi:10.1093/jimb/kuab060)
Supplement: kuab060_Supplemental_File [file kuab060_supplemental_file.docx]

**Supplemental**

Table 1. Fold changes for differentially expressed genes commonly upregulated in *P. destructans* across three treatments with *trans*-2-hexenal (5, 10, or 20 µmol/L) based on a statistical significance of an adjusted p-value < 1^-5^ and a log 2 fold change < 1^-5^ and a log_2_ fold change >1 or <-1. Gene annotations were determined based on the NCBI database and a Blast2GO analysis. Asterisks were used to distinguish genes annotated by one or both databases as follows: no asterisk = Original description from NCBI, One asterisk “*****” = Found in Blast2GO analysis, Two asterisks “******”= Found in both NCBI description and Blast2GO analysis.

| **Gene ID** | **NCBI Description** | **Tran-2-Hexenal Treatment** | | |
| --- | --- | --- | --- | --- |
|  |  | **5 µmol/L** | **10µmol/L** | **20 µmol/L** |
| VC83_00399 | Eukaryotic translation initiation factor 6  (TIF6) ** | 1.7 | 2.6 | 1.9 |
| VC83_02426 | Rab proteins geranylgeranyltransferase component A  (MRS6) ** | 1.4 | 1.7 | 1.4 |
| VC83_02882 | U3 small nucleolar RNA-associated protein 13  (UTP13) ** | 1.6 | 1.9 | 1.8 |
| VC83_03689 | Mitochondrial glycerol-3-phosphate dehydrogenase  (GUT2) ** | 1.4 | 2.0 | 1.6 |
| VC83_04407 | DNA-dependent ATPase of the nucleotide excision repair factor 4 complex  (RAD15) ** | 3.0 | 3.1 | 2.7 |
| VC83_04809 | Translation initiation factor eIF4A  (TIF1) ** | 2.2 | 2.5 | 2.3 |
| VC83_06045 | Protein kinase rio1  (RIO1) ** | 1.7 | 1.8 | 1.6 |
| VC83_06666 | Translational elongation factor EF-1 alpha  (TEF3) ** | 1.3 | 1.4 | 1.6 |
| VC83_06819 | ATP-dependent RNA helicase dbp7  (DBP7) ** | 2.0 | 2.1 | 2.0 |
| VC83_06825 | Ribosome-binding protein  (NMD3) ** | 2.1 | 2.3 | 2.2 |
| VC83_07808 | Mg(2+) transporter  (ALR1) ** | 1.2 | 1.5 | 1.6 |
| VC83_08625 | DEAH-box ATP-dependent RNA helicase prp43  (PRP43) ** | 1.7 | 1.6 | 1.8 |
| VC83_02398 | Thioredoxin-like protein * | 1.6 | 1.8 | 1.4 |
| VC83_02432 | Related to UDP N-acetylglucosamine transporter (Golgi UDP-GlcNAc transporter) * | 1.5 | 1.7 | 1.7 |
| VC83_02682 | Vegetative cell wall protein gp1 * | 2.5 | 2.2 | 2.1 |
| VC83_04527 | Cell wall glucanase protein * | 1.7 | 2.3 | 1.9 |
| VC83_06423 | CDP-alcohol phosphatidyltransferase * | 1.3 | 1.8 | 1.4 |
| VC83_07344 | cobW-domain-containing protein * | 3.1 | 3.1 | 3.9 |
| VC83_07543 | Related to protein FR, involved in hyphal branching * | 1.5 | 1.5 | 1.7 |
| VC83_09405 | P-loop containing nucleoside triphosphate hydrolase * | 3.5 | 3.7 | 3.4 |
| VC83_00494 | Phosphoprotein phosphatase * | 2.8 | 2.8 | 2.1 |
| VC83_00742 | Hypothetical protein VC83_00742 | 2.0 | 2.6 | 1.8 |
| VC83_00877 | Hypothetical protein VC83_00877 | 2.3 | 1.9 | 1.5 |
| VC83_01014 | Hypothetical protein VC83_01014 | 1.4 | 1.8 | 1.8 |
| VC83_01469 | Hypothetical protein VC83_01469 | 1.9 | 2.5 | 2.1 |
| VC83_02188 | Hypothetical protein VC83_02188 | 2.5 | 2.9 | 2.9 |
| VC83_02545 | Hypothetical protein VC83_02545 | 3.7 | 4.0 | 3.9 |
| VC83_02752 | Hypothetical protein VC83_02752 | 1.6 | 1.8 | 1.7 |
| VC83_02862 | Hypothetical protein VC83_02862 | 2.3 | 2.6 | 2.4 |
| VC83_03029 | Hypothetical protein VC83_03029 | 2.2 | 1.7 | 1.8 |
| VC83_03420 | Hypothetical protein VC83_03420 | 2.9 | 3.3 | 2.4 |
| VC83_03740 | Hypothetical protein VC83_03740 | 1.8 | 2.0 | 2.1 |
| VC83_05707 | Hypothetical protein VC83_05707 | 2.0 | 2.9 | 2.5 |
| VC83_05810 | Hypothetical protein VC83_05810 | 2.9 | 3.3 | 2.6 |
| VC83_06295 | Hypothetical protein VC83_06295 | 2.4 | 3.8 | 2.8 |
| VC83_06651 | Hypothetical protein VC83_06651 | 2.4 | 2.1 | 2.7 |
| VC83_06652 | Hypothetical protein VC83_06652 | 2.3 | 2.1 | 2.4 |
| VC83_06756 | Hypothetical protein VC83_06756 | 1.6 | 2.1 | 1.8 |
| VC83_08108 | Hypothetical protein VC83_08108 | 2.1 | 1.9 | 2.8 |
| VC83_08284 | Hypothetical protein VC83_08284 | 1.7 | 1.7 | 1.7 |
| VC83_09335 | Hypothetical protein VC83_09335 | 3.2 | 3.5 | 3.4 |
| VC83_09352 | Hypothetical protein VC83_09352 | 1.9 | 2.5 | 2.1 |
| VC83_09378 | Hypothetical protein VC83_09378 | 2.1 | 2.6 | 2.6 |
| VC83_09443 | Hypothetical protein VC83_09443 | 2.2 | 2.6 | 2.2 |

Table 2. Fold changes of differentially expressed genes commonly downregulated in *P. destructans* across three treatments with *trans*-2-hexenal (5, 10, or 20 µmol/L) based on a statistical significance of an adjusted p-value < 1^-5^ and a log 2 fold change < 1^-5^ and a log_2_ fold change >1 or <-1. Gene annotations were determined based on the NCBI database and a Blast2GO analysis. Asterisks were used to distinguish genes annotated by one or both databases as follows: no asterisk = original description from NCBI, one asterisk “*****” = found in Blast2GO analysis, two asterisks “******”= found in both NCBI description and Blast2GO analysis.

| **Gene_ID** | **NCBI Description** | **Treans-2-Hexenal Treatment** | | |
| --- | --- | --- | --- | --- |
|  |  | **5 µmol/L** | **10µmol/L** | **20 µmol/L** |
| VC83_00883 | Methylglyoxal reductase (NADPH-dependent) gre2  (GRE2) ** | -1.6 | -2.2 | -2.0 |
| VC83_06276 | Vesicle formation at the endoplasmic reticulum  (SED4) ** | -2.8 | -3.5 | -4.2 |
| VC83_04892 | Subtilisin-like protease 1  (SP1) ** | -3.6 | -3.7 | -3.9 |
| VC83_06822 | Phosphomevalonate kinase  (ERG8) ** | -1.4 | -1.9 | -1.6 |
| VC83_07077 | Superoxide dismutase  (SOD1) ** | -1.9 | -2.1 | -2.4 |
| VC83_08761 | Glyceraldehyde-3-phosphate dehydrogenase 1  (GAP1_3) ** | -2.2 | -3.0 | -2.7 |
| VC83_00241 | Concanavalin A-like lectin/glucanase * | -2.4 | -4.2 | -4.0 |
| VC83_01030 | NTF2-like protein * | -1.3 | -1.6 | -1.3 |
| VC83_01564 | Guanyl-specific ribonuclease f1 * | -2.2 | -3.7 | -3.6 |
| VC83_01724 | Putative oligopeptide transporter * | -2.0 | -3.0 | -2.8 |
| VC83_02203 | EF-hand calcium-binding domain protein * | -1.8 | -2.3 | -1.8 |
| VC83_02268 | MFS general substrate transporter * | -3.0 | -4.1 | -3.8 |
| VC83_02906 | MFS general substrate transporter * | -3.3 | -5.9 | -6.0 |
| VC83_03247 | Peptidase S28 * | -2.8 | -4.4 | -4.3 |
| VC83_04545 | Amino acid transporter * | -1.9 | -2.7 | -2.3 |
| VC83_06026 | Amino acid permease * | -1.6 | -2.3 | -1.9 |
| VC83_07035 | Putative phosphate transport protein * | -1.6 | -1.8 | -1.8 |
| VC83_07149 | Related to high-affinity iron permease * | -1.7 | -2.9 | -2.0 |
| VC83_07171 | Primary-amine oxidase * | -2.1 | -2.7 | -2.6 |
| VC83_07336 | Oligopeptide transporter * | -2.0 | -2.3 | -2.5 |
| VC83_00345 | Hypothetical protein VC83_00345 | -2.7 | -4.6 | -5.3 |
| VC83_01565 | Hypothetical protein VC83_01565 | -1.6 | -2.2 | -2.0 |
| VC83_02181 | Hypothetical protein VC83_02181 | -2.1 | -2.4 | -2.7 |
| VC83_02269 | Hypothetical protein VC83_02269 | -3.0 | -3.6 | -3.6 |
| VC83_02402 | Hypothetical protein VC83_02402 | -1.4 | -2.0 | -1.7 |
| VC83_06127 | Hypothetical protein VC83_06127 | -2.4 | -2.7 | -2.8 |
| VC83_06378 | Hypothetical protein VC83_06378 | -1.9 | -3.1 | -2.3 |
| VC83_06475 | Hypothetical protein VC83_06475 | -2.2 | -2.0 | -1.9 |
| VC83_06806 | Hypothetical protein VC83_06806 | -2.5 | -3.5 | -2.8 |
| VC83_07272 | Hypothetical protein VC83_07272 | -1.6 | -2.2 | -1.7 |
